# (μ-4,4′-Bipyridine-κ^2^ *N*:*N*′)bis­[bis­(*N*,*N*-dimethyl­dithio­carbamato-κ^2^ *S*,*S*′)zinc(II)]

**DOI:** 10.1107/S1600536810042650

**Published:** 2010-10-23

**Authors:** Mei-Qin Zha, Xing Li, Yue Bing, Yue Lu

**Affiliations:** a Faculty of Materials Science and Chemical Engineering, Ningbo University, Ningbo Zhejiang 315211, People’s Republic of China

## Abstract

The title dinuclear Zn^II^ complex, [Zn_2_(C_3_H_6_NS_2_)_4_(C_10_H_8_N_2_)], is centrosymmetric; the mid-point of the C—C bond linking the two pyridine rings is located on an inversion center. The pyridine N atom coordinates to the Zn^II^ cation, which is also chelated by two dimethyl­dithio­carbamate anions, giving a trigonal-bipyramidal ZnNS_4_ geometry. Weak inter­molecular C—H⋯S hydrogen bonding is present in the crystal structure.

## Related literature

Dialkyl­dithio­carbamates have strong metal-binding properties as well as biological functions, see: Jian *et al.* (2002 ▶); Arora *et al.* (2003 ▶); Hogarth & Richards (2006 ▶). For related zinc(II) dithio­carbamate compounds, see: Lai *et al.* (2002 ▶); Chen *et al.* (2006 ▶); Benson *et al.* (2007 ▶).
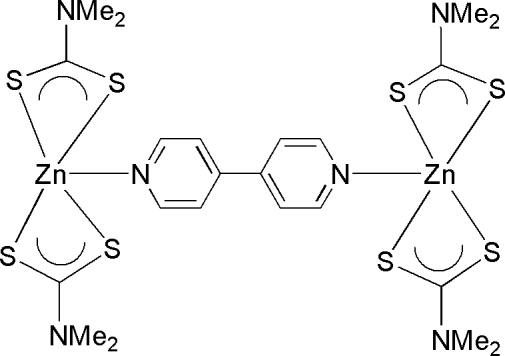


## Experimental

### 

#### Crystal data

[Zn_2_(C_3_H_6_NS_2_)_4_(C_10_H_8_N_2_)]
*M* *_r_* = 767.76
Monoclinic, 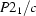

*a* = 8.0490 (8) Å
*b* = 13.8770 (14) Å
*c* = 14.8134 (14) Å
β = 100.070 (1)°
*V* = 1629.1 (3) Å^3^
*Z* = 2
Mo *K*α radiation
μ = 2.01 mm^−1^
*T* = 173 K
0.34 × 0.26 × 0.13 mm

#### Data collection

Bruker SMART 1000 CCD area-detector diffractometer
Absorption correction: multi-scan (*SADABS*; Bruker, 2001 ▶) *T*_min_ = 0.540, *T*_max_ = 0.770
14059 measured reflections
3754 independent reflections
3205 reflections with *I* > 2σ(*I*)
*R*_int_ = 0.044

#### Refinement

*R*[*F* ^2^ > 2σ(*F* ^2^)] = 0.040
*wR*(*F* ^2^) = 0.101
*S* = 1.04
3754 reflections
170 parameters
H-atom parameters constrained
Δρ_max_ = 1.21 e Å^−3^
Δρ_min_ = −1.95 e Å^−3^

### 

Data collection: *SMART* (Bruker, 2001 ▶); cell refinement: *SAINT* (Bruker, 2001 ▶); data reduction: *SAINT*; program(s) used to solve structure: *SHELXTL* (Sheldrick, 2008 ▶); program(s) used to refine structure: *SHELXTL*; molecular graphics: *SHELXTL*; software used to prepare material for publication: *SHELXTL*.

## Supplementary Material

Crystal structure: contains datablocks I, global. DOI: 10.1107/S1600536810042650/xu5042sup1.cif

Structure factors: contains datablocks I. DOI: 10.1107/S1600536810042650/xu5042Isup2.hkl

Additional supplementary materials:  crystallographic information; 3D view; checkCIF report

## Figures and Tables

**Table 1:** Selected bond lengths (Å)

Zn1—N1	2.064 (2)
Zn1—S1	2.5909 (9)
Zn1—S2	2.3488 (9)
Zn1—S3	2.3495 (8)
Zn1—S4	2.6239 (9)

**Table 2:** Hydrogen-bond geometry (Å, °)

*D*—H⋯*A*	*D*—H	H⋯*A*	*D*⋯*A*	*D*—H⋯*A*
C4—H4*A*⋯S3^i^	0.95	2.86	3.782 (3)	164

Symmetry code: (i) 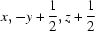.

**Fig. 1.**
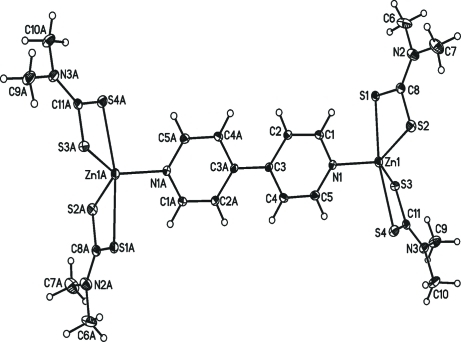
The molcular structure of (I) showing displacement ellipsoids at the 30% probability level.

**Fig. 2.**
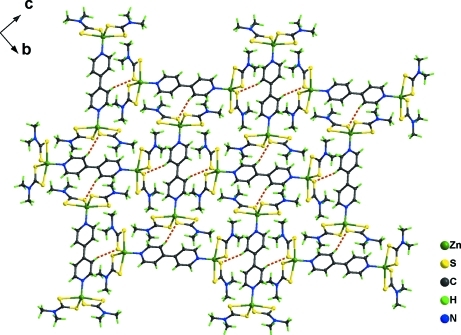
Two-dimensional supramolecular framework for (I) by C—H···S interactions (orange dashed lines).

**Fig. 3.**
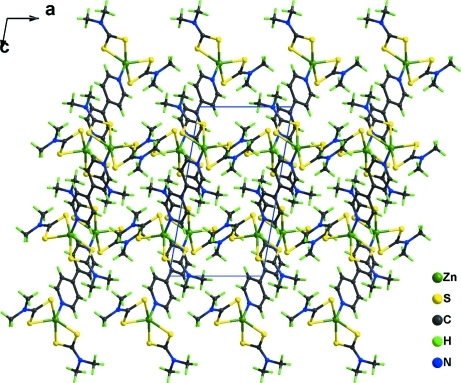
Unit-cell contents for (I) viewed in projection down the b axis.

## supplementary crystallographic information

### Comment

Dialkyldithiocarbamates, (R_2_dtc) (where R is an alkyl group such as methyl, ethyl or propyl), have strong metal-binding properties as well as biological functions (Jian *et al.*, 2002; Arora *et al.*, 2003; Hogarth & Richards, 2006). Crystal engineering studies of zinc(II) dithiocarbamates (^-^S_2_CNR_2_) are less well developed (Lai *et al.*, 2002; Chen *et al.*, 2006; Benson *et al.*, 2007), this is likely due to the stronger chelating ability of the dithiocarbamate ligand which tends to preclude incorporation of multiple bridging ligands within the Zn atom coordination sphere. Here, we report the crystal structure of the title zinc complex with 4,4'-bipy and dimethyldithiocarbamate (Me_2_dtc), [Zn_2_(C_3_H_6_NS_2_)_4_(C_10_H_8_N_2_)], (I).

Complex (I) is a binuclear structure, in which Zn1A is symmetrical component related by Zn1 (Symmetry code: -*x*, *y* + 1/2, -*z* + 3/2), and the two Zn^2+^ ions possess the same coordination environment (Fig. 1). The Zn^2+^ ion adopts a distorted square-pyramidal coordination geometry comprising two S,*S*'- bidentate dimethyldithiocarbamate (Me_2_dtc) ligands, one N atom from 4,4'-bipy ligand, the N atom in the apical site, Zn—O distances ranging from 2.349 to 2.624 Å and Cd –N distance being 2.064 Å. In the crystal, the molecules are generate to a one-dimensional chain, which is further extended into two-dimensional supramolecular network *via* weak C—H···S contacts (Fig. 2), and finally assembled into three-dimensional supramolecular network by C—H···π interactions (Fig. 3).

### Experimental

Tetramethylthiuram monosulfide (5.0 mg, 0.024 mmol) dissolved in *N*,*N*-dimethylformamide (DMF) (2 ml) was mixed with a DMF solution (1 ml) of 4,4'-bipy (2.38 mg, 0.012 mmol) and stirred for 20 min at room temperature. A DMF solution (0.2 ml) of Zn(NO_3_)_2_.6H_2_O (3.57 mg, 0.012 mmol) was then added dropwise and the mixture was allowed to react for 15 min. The solution was left at room temperature to allow slow evaporation. After a few days, pale yellow block crystals of (I) were obtained from the mother liquor.

### Refinement

H atoms were placed in calculated positions and treated using a riding-model approximation with C–H = 0.93–0.98 Å and U_iso_(H) = 1.2U_eq_(C) or 1.5U_eq_(C).

### Crystal data

**Table:**

[Zn_2_(C_3_H_6_NS_2_)_4_(C_10_H_8_N_2_)]	*F*(000) = 788
*M**_r_* = 767.76	*D*_x_ = 1.565 Mg m^−^^3^
Monoclinic, *P*2_1_/*c*	Mo *K*α radiation, λ = 0.71073 Å
Hall symbol: -P 2ybc	Cell parameters from 14059 reflections
*a* = 8.0490 (8) Å	θ = 2.0–27.5°
*b* = 13.8770 (14) Å	µ = 2.01 mm^−^^1^
*c* = 14.8134 (14) Å	*T* = 173 K
β = 100.070 (1)°	Block, yellow
*V* = 1629.1 (3) Å^3^	0.34 × 0.26 × 0.13 mm
*Z* = 2	

### Data collection

**Table:**

Bruker SMART 1000 CCD area-detector diffractometer	3754 independent reflections
Radiation source: fine-focus sealed tube	3205 reflections with *I* > 2σ(*I*)
graphite	*R*_int_ = 0.044
φ and ω scans	θ_max_ = 27.5°, θ_min_ = 2.0°
Absorption correction: multi-scan (*SADABS*; Bruker, 2001)	*h* = −10→10
*T*_min_ = 0.540, *T*_max_ = 0.770	*k* = −18→15
14059 measured reflections	*l* = −18→19

### Refinement

**Table:**

Refinement on *F*^2^	Primary atom site location: structure-invariant direct methods
Least-squares matrix: full	Secondary atom site location: difference Fourier map
*R*[*F*^2^ > 2σ(*F*^2^)] = 0.040	Hydrogen site location: inferred from neighbouring sites
*wR*(*F*^2^) = 0.101	H-atom parameters constrained
*S* = 1.04	*w* = 1/[σ^2^(*F*_o_^2^) + (0.0365*P*)^2^ + 3.8184*P*] where *P* = (*F*_o_^2^ + 2*F*_c_^2^)/3
3754 reflections	(Δ/σ)_max_ < 0.001
170 parameters	Δρ_max_ = 1.21 e Å^−^^3^
0 restraints	Δρ_min_ = −1.95 e Å^−^^3^

### Special details

**Table:**

Geometry. All e.s.d.'s (except the e.s.d. in the dihedral angle between two l.s. planes) are estimated using the full covariance matrix. The cell e.s.d.'s are taken into account individually in the estimation of e.s.d.'s in distances, angles and torsion angles; correlations between e.s.d.'s in cell parameters are only used when they are defined by crystal symmetry. An approximate (isotropic) treatment of cell e.s.d.'s is used for estimating e.s.d.'s involving l.s. planes.
Refinement. Refinement of *F*^2^ against ALL reflections. The weighted *R*-factor *wR* and goodness of fit *S* are based on *F*^2^, conventional *R*-factors *R* are based on *F*, with *F* set to zero for negative *F*^2^. The threshold expression of *F*^2^ > σ(*F*^2^) is used only for calculating *R*-factors(gt) *etc*. and is not relevant to the choice of reflections for refinement. *R*-factors based on *F*^2^ are statistically about twice as large as those based on *F*, and *R*- factors based on ALL data will be even larger.

### Fractional atomic coordinates and isotropic or equivalent isotropic displacement parameters (Å^2^)

**Table:**

	*x*	*y*	*z*	*U*_iso_*/*U*_eq_	
Zn1	0.13314 (4)	0.27435 (3)	0.24330 (2)	0.02256 (11)	
S1	0.08181 (9)	0.16003 (5)	0.10290 (5)	0.02256 (11)	
S2	−0.12799 (10)	0.31820 (6)	0.15575 (6)	0.02636 (18)	
S3	0.39861 (9)	0.33093 (6)	0.22187 (5)	0.02260 (17)	
S4	0.21808 (10)	0.41611 (6)	0.35942 (6)	0.02694 (18)	
C1	−0.0291 (4)	0.1060 (2)	0.3154 (2)	0.0286 (7)	
H1A	−0.0887	0.1049	0.2541	0.034*	
C2	−0.0689 (4)	0.0385 (2)	0.3764 (2)	0.0292 (7)	
H2A	−0.1562	−0.0069	0.3570	0.035*	
C3	0.0183 (4)	0.0363 (2)	0.46632 (19)	0.0183 (6)	
C4	0.1429 (4)	0.1065 (2)	0.4901 (2)	0.0236 (6)	
H4A	0.2065	0.1084	0.5504	0.028*	
C5	0.1731 (4)	0.1731 (2)	0.4255 (2)	0.0235 (6)	
H5A	0.2568	0.2209	0.4434	0.028*	
C6	−0.1905 (5)	0.1389 (3)	−0.0636 (3)	0.0444 (10)	
H6A	−0.0751	0.1136	−0.0503	0.067*	
H6B	−0.2123	0.1658	−0.1257	0.067*	
H6C	−0.2706	0.0867	−0.0591	0.067*	
C7	−0.3640 (5)	0.2736 (4)	−0.0185 (3)	0.0495 (11)	
H7A	−0.4042	0.2895	0.0385	0.074*	
H7B	−0.4514	0.2375	−0.0591	0.074*	
H7C	−0.3387	0.3332	−0.0489	0.074*	
C8	−0.0971 (4)	0.2285 (2)	0.0785 (2)	0.0230 (6)	
C9	0.6095 (5)	0.5119 (3)	0.2449 (3)	0.0372 (8)	
H9A	0.5971	0.4609	0.1985	0.056*	
H9B	0.7211	0.5072	0.2836	0.056*	
H9C	0.5976	0.5750	0.2147	0.056*	
C10	0.4667 (5)	0.5812 (3)	0.3652 (3)	0.0362 (8)	
H10A	0.3668	0.5723	0.3939	0.054*	
H10B	0.4570	0.6421	0.3312	0.054*	
H10C	0.5680	0.5825	0.4127	0.054*	
C11	0.3763 (4)	0.4248 (2)	0.2965 (2)	0.0206 (6)	
N1	0.0906 (3)	0.17348 (17)	0.33906 (17)	0.0203 (5)	
N2	−0.2105 (3)	0.2147 (2)	0.0027 (2)	0.0331 (7)	
N3	0.4789 (3)	0.50091 (19)	0.30182 (18)	0.0255 (6)	

### Atomic displacement parameters (Å^2^)

**Table:**

	*U*^11^	*U*^22^	*U*^33^	*U*^12^	*U*^13^	*U*^23^
Zn1	0.02293 (17)	0.02255 (18)	0.02164 (18)	−0.00291 (12)	0.00229 (12)	0.00392 (12)
S1	0.02293 (17)	0.02255 (18)	0.02164 (18)	−0.00291 (12)	0.00229 (12)	0.00392 (12)
S2	0.0227 (4)	0.0276 (4)	0.0287 (4)	0.0040 (3)	0.0042 (3)	0.0005 (3)
S3	0.0212 (3)	0.0237 (4)	0.0230 (4)	−0.0016 (3)	0.0042 (3)	−0.0034 (3)
S4	0.0300 (4)	0.0249 (4)	0.0284 (4)	−0.0040 (3)	0.0119 (3)	−0.0024 (3)
C1	0.0389 (18)	0.0250 (16)	0.0183 (15)	−0.0113 (13)	−0.0045 (13)	0.0017 (12)
C2	0.0391 (18)	0.0238 (16)	0.0216 (15)	−0.0152 (14)	−0.0032 (13)	0.0036 (12)
C3	0.0251 (14)	0.0133 (13)	0.0171 (13)	−0.0003 (11)	0.0055 (11)	−0.0020 (11)
C4	0.0269 (15)	0.0259 (16)	0.0167 (14)	−0.0071 (12)	0.0002 (11)	0.0008 (12)
C5	0.0240 (14)	0.0245 (16)	0.0217 (15)	−0.0074 (12)	0.0032 (12)	−0.0016 (12)
C6	0.039 (2)	0.065 (3)	0.0268 (18)	−0.0130 (19)	0.0011 (15)	−0.0150 (18)
C7	0.0289 (19)	0.072 (3)	0.043 (2)	0.0039 (19)	−0.0056 (17)	0.006 (2)
C8	0.0222 (14)	0.0263 (16)	0.0213 (15)	−0.0054 (12)	0.0057 (11)	0.0024 (12)
C9	0.0364 (18)	0.036 (2)	0.043 (2)	−0.0150 (15)	0.0175 (16)	−0.0078 (16)
C10	0.042 (2)	0.0242 (17)	0.045 (2)	−0.0088 (15)	0.0146 (16)	−0.0106 (15)
C11	0.0202 (13)	0.0212 (14)	0.0190 (14)	0.0016 (11)	−0.0004 (11)	0.0035 (11)
N1	0.0254 (12)	0.0168 (12)	0.0186 (12)	−0.0027 (10)	0.0034 (10)	0.0014 (9)
N2	0.0236 (13)	0.0464 (18)	0.0279 (15)	−0.0042 (12)	0.0005 (11)	0.0005 (13)
N3	0.0268 (13)	0.0219 (13)	0.0290 (14)	−0.0045 (10)	0.0082 (11)	−0.0022 (11)

### Geometric parameters (Å, °)

**Table:**

Zn1—N1	2.064 (2)	C5—H5A	0.9500
Zn1—S1	2.5909 (9)	C6—N2	1.467 (5)
Zn1—S2	2.3488 (9)	C6—H6A	0.9800
Zn1—S3	2.3495 (8)	C6—H6B	0.9800
Zn1—S4	2.6239 (9)	C6—H6C	0.9800
S1—C8	1.710 (3)	C7—N2	1.468 (5)
S2—C8	1.738 (3)	C7—H7A	0.9800
S3—C11	1.738 (3)	C7—H7B	0.9800
S4—C11	1.708 (3)	C7—H7C	0.9800
C1—N1	1.345 (4)	C8—N2	1.331 (4)
C1—C2	1.378 (4)	C9—N3	1.466 (4)
C1—H1A	0.9500	C9—H9A	0.9800
C2—C3	1.393 (4)	C9—H9B	0.9800
C2—H2A	0.9500	C9—H9C	0.9800
C3—C4	1.398 (4)	C10—N3	1.471 (4)
C3—C3^i^	1.483 (6)	C10—H10A	0.9800
C4—C5	1.383 (4)	C10—H10B	0.9800
C4—H4A	0.9500	C10—H10C	0.9800
C5—N1	1.335 (4)	C11—N3	1.334 (4)
			
N1—Zn1—S2	108.37 (7)	H6B—C6—H6C	109.5
N1—Zn1—S3	125.72 (7)	N2—C7—H7A	109.5
S2—Zn1—S3	125.87 (3)	N2—C7—H7B	109.5
N1—Zn1—S1	96.55 (7)	H7A—C7—H7B	109.5
S2—Zn1—S1	73.36 (3)	N2—C7—H7C	109.5
S3—Zn1—S1	96.74 (3)	H7A—C7—H7C	109.5
N1—Zn1—S4	96.52 (7)	H7B—C7—H7C	109.5
S2—Zn1—S4	105.85 (3)	N2—C8—S1	121.7 (3)
S3—Zn1—S4	72.49 (3)	N2—C8—S2	120.2 (3)
S1—Zn1—S4	166.41 (3)	S1—C8—S2	118.10 (18)
C8—S1—Zn1	80.77 (11)	N3—C9—H9A	109.5
C8—S2—Zn1	87.75 (11)	N3—C9—H9B	109.5
C11—S3—Zn1	88.10 (10)	H9A—C9—H9B	109.5
C11—S4—Zn1	80.15 (10)	N3—C9—H9C	109.5
N1—C1—C2	122.7 (3)	H9A—C9—H9C	109.5
N1—C1—H1A	118.6	H9B—C9—H9C	109.5
C2—C1—H1A	118.6	N3—C10—H10A	109.5
C1—C2—C3	120.4 (3)	N3—C10—H10B	109.5
C1—C2—H2A	119.8	H10A—C10—H10B	109.5
C3—C2—H2A	119.8	N3—C10—H10C	109.5
C2—C3—C4	116.4 (3)	H10A—C10—H10C	109.5
C2—C3—C3^i^	122.1 (3)	H10B—C10—H10C	109.5
C4—C3—C3^i^	121.5 (3)	N3—C11—S4	122.4 (2)
C5—C4—C3	119.8 (3)	N3—C11—S3	119.9 (2)
C5—C4—H4A	120.1	S4—C11—S3	117.64 (17)
C3—C4—H4A	120.1	C5—N1—C1	117.4 (3)
N1—C5—C4	123.2 (3)	C5—N1—Zn1	123.2 (2)
N1—C5—H5A	118.4	C1—N1—Zn1	119.4 (2)
C4—C5—H5A	118.4	C8—N2—C6	121.9 (3)
N2—C6—H6A	109.5	C8—N2—C7	121.8 (3)
N2—C6—H6B	109.5	C6—N2—C7	116.3 (3)
H6A—C6—H6B	109.5	C11—N3—C9	123.2 (3)
N2—C6—H6C	109.5	C11—N3—C10	121.8 (3)
H6A—C6—H6C	109.5	C9—N3—C10	115.0 (3)

Symmetry codes: (i) −*x*, −*y*, −*z*+1.

### Hydrogen-bond geometry (Å, °)

**Table:**

*D*—H···*A*	*D*—H	H···*A*	*D*···*A*	*D*—H···*A*
C4—H4A···S3^ii^	0.95	2.86	3.782 (3)	164

Symmetry codes: (ii) *x*, −*y*+1/2, *z*+1/2.
